# Exploring Patterns of Men’s Self-Reported Sexual Behaviours, Feelings, and Interests Towards Children

**DOI:** 10.1177/08862605251403602

**Published:** 2026-03-03

**Authors:** Tyson Whitten, Michael Salter, Delanie Woodlock

**Affiliations:** 1 School of Social Sciences, University of New South Wales, Sydney, Australia

**Keywords:** sexual abuse, child maltreatment, offenders/perpetrators, prevalence

## Abstract

Child sexual exploitation and abuse (CSEA) is prevalent worldwide. Yet, knowledge about potential perpetrators in the community is constrained by reliance on justice-involved and clinical samples, which limits external validity and obscures undetected behaviour. This study estimates population-level prevalence, demographic correlates, and co-endorsement patterns of men’s self-reported sexual feelings, interests, and behaviours towards children. We analyse an anonymous online survey of 4,918 adult men quota-matched and weighted to national populations in Australia, the United Kingdom, and the United States. In pooled analyses, 8.0% reported sexual feelings towards children, 7.4% would likely have sexual contact with a child if undetected, 5.5% to 5.7% would watch child sexual abuse material or a webcam show, and 2.4% to 4.7% reporting engagement in online or contact offending. Prevalence estimates were consistently higher in the United States than in Australia and the United Kingdom. Age distributions generally showed peaks in early adulthood with subsequent decline, alongside later-life upticks for selected outcomes. Sociodemographic indicators linked to trust or access (higher income, being partnered, employment, university education, children in the household, and working with children) were consistently associated with multiple outcomes, with the largest effect sizes for men who live or work with children. Overlap analyses and a nodewise LASSO-based Ising network indicated coherent clusters (online behaviours, contact behaviour, and interest) with strong within-cluster and bridging connections. Findings support tiered prevention that distinguishes interest from behaviour, age-responsive strategies, and strengthened safeguards for child-contact roles, while providing cross-national baselines to inform surveillance, resource allocation, and targeted intervention.

## Introduction

Child sexual exploitation and abuse (CSEA) refers to any act that involves a child in sexual activity that they do not understand, do not or cannot consent to, and that breaches social and legal norms. CSEA is a pervasive global problem with profound and enduring harms for victims, families, and communities (United Nations Children’s Fund, 2025; World Health Organization, n.d.), including increasing victims’ risks of health and socio-economic adversity and imposing substantial economic costs on society (Akinyemi et al., 2025; Hailes et al., 2019; Henkhaus, 2022). Effective prevention depends on a clear understanding of potential perpetrators: who they are, the range of sexual feelings and behaviours they report, and how these experiences cluster. Yet, much of what is known about offenders is inferred from victimisation studies, justice-system data, and clinical or forensic samples (Lussier et al., 2020). These data are essential for response and treatment but undercount undetected offending, overrepresent identified subgroups, and can obscure hidden behaviours and interests in the community (Drury et al., 2020; Lussier et al., 2020; Neutze et al., 2012). Anonymous, representative community-based surveys are a necessary complement to existing data sources that help capture information on undetected CSEA offenders and provide more accurate population-level estimates. The present study contributes to understanding this cohort of offenders by examining the prevalence, demographic correlates, and overlap of men’s self-reported sexual feelings, interests, and behaviours towards children. The focus on males reflects their overwhelming predominance among identified child-sex offenders, while recognising that female offending occurs but may involve distinct risk profiles and contexts (Burgess-Proctor et al., 2017).

## Literature Review

Community-based research indicates that a non-trivial minority of adult men endorse sexual feelings, interests, or behaviours involving children, though estimates differ substantially by sampling frame, measurement, and context (Savoie et al., 2021). Across surveys, indicators of sexual feelings (i.e. attraction and arousal) and interest – defined as a curiosity or motivation towards CSEA-related behaviours (e.g. hypothetical willingness to view child sexual abuse material (CSAM) under guaranteed anonymity, or sexual contact if undetected) – are consistently more prevalent than enacted behaviours such as consumption of CSAM, sexual communication with a minor, or contact offending (Bártová et al., 2021; Dombert et al., 2016; Joyal & Carpentier, 2017; Ó Ciardha et al., 2022; Seto et al., 2015). Prevalence estimates are typically higher among younger male cohorts, and comparatively rarer in representative adult samples. Overall, the literature indicates a descending prevalence gradient, from feelings to interests to behaviour, with a strong sensitivity to item wording, assurances of anonymity, and sample representativeness (Savoie et al., 2021).

This prevalence pattern is illustrated across jurisdictions. For example, Dombert et al. (2016) reported that 4.1% of German men endorsed sexual fantasies involving prepubescent children, 2.4% reported consuming child pornography, and 1.5% indicated physical sexual contact. In the Czech Republic, Bártová et al. (2021) found that 5.1% of men indicated sexual arousal towards prepubescent children and 25.9% towards pubescent children, with 3.1% and 9.7%, respectively, reporting consumption of pornography depicting these groups. In Canada, Joyal and Carpentier (2017) observed that 1.1% of men expressed sexual interest in prepubescent children, while 0.6% reported related behaviours. Among Swedish males aged 17 to 20 years, Seto et al. (2015) reported that 4.2% had viewed child pornography and 9.9% were likely or very likely to have sexual contact with a child if they would not get caught. Finally, an international online survey found that 16.8% of men reported sexual interest in children, 10.9% reported a willingness for sexual contact with children, and 3.3% and 2.9% reported CSAM related or contact offending, respectively (Ó Ciardha et al., 2022).

A second strand of community-based evidence concerns the characteristics of undetected offenders and men who endorse sexual interests in children. Many men who sexually offend against children remain undetected (Brown, 2023; Krone et al., 2017). Because the risk of detection increases with opportunities to offend, it is possible that undetected offenders disproportionately occupy roles and life circumstances associated with trust and access to children. Consistent with this hypothesis, several studies report that, relative to non-offending men with similar interests, undetected offenders tend to be older, more likely to be partnered, employed, better educated, and report higher income or access to children through work or in the household (Bailey et al., 2016; Brown, 2023; Cohen et al., 2018; Neutze et al., 2012). There is also some evidence of age-related heterogeneity, with involvement in CSEA disproportionately higher among men under 35 and over 55 years (Napier et al., 2024; Seigfried-Spellar, 2014). Population surveys focused on technology-facilitated CSEA identify profiles that may differ from contact-offending profiles on average, skewing younger and more educated, while still overlapping substantially on psychosocial correlates (Babchishin et al., 2015; Faust et al., 2015).

A robust finding across studies is the overlap between sexual feelings, interests, and behaviours towards children, as well as the link between online and offline offending. Measures of sexual arousal and fantasy are positively associated with self-reported online and offline offending (Dombert et al., 2016; Joyal & Carpentier, 2017; Seto et al., 2021). Online behaviours, including CSAM consumption and livestreaming CSEA, are significantly more likely to co-occur with both sexual interests and contact offending (Bártová et al., 2021; Maimone et al., 2024; Ray et al., 2014). These patterns are consistent with models in which sexual preference, opportunity, and self-regulation jointly shape offending pathways, and in which online activities can serve as both a marker of risk and a mechanism that normalises or escalates behaviour for some men (Seto, 2019; Wortley & Smallbone, 2014). Nonetheless, many men reporting sexual feelings and interests towards children deny engagement in offending behaviour, illustrating the heterogeneity in these patterns and the potential for prevention (Cantor & McPhail, 2016).

Despite important advances by the extant literature, several gaps remain. First, few studies have fielded large, representative, community-based samples, and none have done so across multiple countries, limiting external validity and comparability (Savoie et al., 2021). Second, evidence on the demographic correlates of men’s sexual feelings, interests, and behaviours towards children is fragmented: Findings come from separate studies that examine one domain at a time, and, to our knowledge, no single study has evaluated demographic indicators of social trust across all three domains within the same sample. Third, most work examines pairwise associations rather than mapping patterns of co-occurrence across multiple indices, leaving uncertainty about how different interests and behaviours cluster. Addressing these gaps requires population-based data, standardised measurement across countries, and analyses that quantify both prevalence and co-endorsement structures.

## The Current Study

In this exploratory study, we estimate the prevalence, age distribution, and demographic correlates of responses to 10 self‑report items capturing men’s sexual behaviours, feelings, and interests towards children. We then examine overlap and co‑endorsement across items to characterise how indicators cluster. Data are drawn from an anonymous online survey of 4,918 adult men quota‑matched and weighted to approximate the Australian, U.K., and U.S. male populations. By providing population‑based estimates across three countries and analysing item‑level associations, we address limitations of prior research that relied on single‑country convenience samples, forensic populations, or pairwise analyses. This exploratory design is warranted because cross‑country, population‑based benchmarks for discrete indicators are scarce; item‑specific estimates and correlates offer actionable baselines for policy (e.g. safeguarding standards and screening in child‑contact roles), practice (e.g. triage, referral, and low‑barrier support for interest versus behaviour), and surveillance (e.g. monitoring change over time). Identifying which indicators are most common, and among whom, helps pinpoint prevention targets and provides a shared starting point for future research to build upon.

## Methods

### Data

An online survey was conducted examining the prevalence and factors associated with men’s sexual attitudes, feelings, and behaviours towards children. Data were drawn from three quota-based samples of men aged 18 years or over comparable to the Australian, U.K., and U.S. male populations in terms of age, residential region, annual household income, and educational attainment. Survey recruitment and administration were conducted by CloudResearch (https://www.cloudresearch.com) using the Prime Panels platform. Invitations were sent based on demographic profiles, and participants were compensated for completing the survey. The survey was reviewed by a project advisory group, which included representatives from law enforcement, financial intelligence agencies, government departments, and mental health support services. Surveys were administered from November to December 2022. Ethics approval for this study was provided by the University of New South Wales (HC220317) prior to data collection.

The study invitation described the research as a survey on men’s attitudes and behaviours related to online child abuse. It specified that participants had to be men aged 18 or older and outlined that the survey would cover areas such as demographics, online activity, mental health, pornography use, childhood abuse, and views on child sexual abuse. The invitation emphasised that participation was voluntary, anonymous, and confidential and that the survey would take about 15 to 20 min. CloudResearch’s Sentry® system was used to screen out inattentive, dishonest, or duplicate respondents, as well as those using VPNs or bots, with additional attention checks embedded in the survey to filter out noncompliant participants.

Of the 7,343 individuals who consented to participate (Australia = 2,703; United Kingdom = 2,243; United States = 2,397), 6,577 completed the survey (retention rates: Australia = 92.2%, United Kingdom = 87.4%, United States = 88.6%). After excluding participants who were not male at birth (*n* = 198), did not identify as male (*n* = 178), failed attention checks (*n* = 1,506), or admitted dishonest responses (*n* = 115), and removing 68 with missing key demographic data, the final analytical sample comprised 4,918 men (Australia = 1,939; United Kingdom = 1,506; United States = 1,473). For a detailed description of the study methodology, see Whitten et al. (in press).

### Measures

#### Sexual Behaviours, Feelings, and Interest Towards Children

Participants reported their engagement (0 = *no, maybe, or unsure*; 1 = *yes*) in the following behaviours anytime since their 18th birthday: (1) “*knowingly and deliberately viewed pornographic material containing people below the age of 18*”; (2) “*had sex or sexual contact with a person below the age of 18*”; (3) “*flirted or had sexual conversations with a person below the age of 18 online*”; (4) “*webcammed in a sexual way with a person below the age of 18*”; (5) “*paid for online sexual interactions, images or videos involving a person below the age of 18*”. Men also indicated the following: (6) “*if you could be sure that you were anonymous online, would you watch pornographic material of people below the age of 18*?”; (7) “*if you were offered a webcam sex show of someone under the age of 18, would you watch it?”*; (8) “*I have sexual feelings towards people below the age of 18*”; and (9) “*I have concerns about my sexual feelings towards people below the age of 18*”. Finally, participants were asked (10) *“what is the likelihood that you would have sexual contact with a child if you were certain that no one would find out and that you would not be punished*?” separately for ages (a) between 12 and 14 years, (b) between 10 and 12 years, and (c) under 10 years; a composite variable was created indicating if participants responded “*very likely*” or “*definitely*” (vs. never, unlikely, or maybe) for any of the three age brackets.

#### Demographic Factors

Age (1 = *18–24 years*; 2 = *25–34 years*; 3 = *35–44 years*; 4 = *45–54 years*; 5 = *55–64 years*; 6 = *65 years and older*), current relationship status (1 = *married or de facto relationship*; 0 = *widowed, separated, divorced, or never married*), educational attainment (1 = *bachelor’s degree or higher*; 0 = *below high school, high school, vocational or diploma*), high annual household income before taxes (1 = *US$100,000 equivalent or more*; 0 = *less than US$100,000 equivalent*), employment status in the last 3 months (1 = *full time, part time, or casual employment*; 0 = *not currently working*), children living in the household (1 = *one or more children*; 0 = *no children*), and work currently involves contact with children under 18 (0 = *no or not working*; 1 = *yes*).

### Statistical Analyses

Iterative proportional fitting was used to weigh responses by age, annual household income, race, educational attainment, marital status, and workforce participation, based on national census data. All analyses were calculated using survey weights and robust standard errors (Lumley, 2004). Prevalence estimates with 99% confidence intervals (CI) were presented for each sexual behaviour, feeling, and interest item for the pooled sample (*N* = 4,918) and stratified by country and age bracket. Between-country differences were detected using the adjusted *F* statistic variant of the second-order Rao–Scott chi-squared test. A series of logistic regression analyses were conducted to examine the unadjusted association between the sexual behaviour, feeling, and interest items and six demographic indicators of socio-economic status and trust: high annual household income, married or de facto relationship, employed, university educated, one or more children living in the household, and work involving contact with children. Cross-tabulations based on row proportions were used to explore the overlap across the sexual behaviours, feelings, and interest items; these were calculated alongside unadjusted odds ratios (ORs) indicating the likelihood of item co-endorsement.

Finally, a conditional dependence network was estimated using a nodewise LASSO Ising to explore patterns of co-endorsement among the sexual behaviours, feelings, and interest items. LASSO imposes a penalty on the regression coefficients, preventing overfitting and simplifying the model by shrinking irrelevant coefficients to zero, resulting in a sparse subset of the most predictive variables (Ajana et al., 2019). For each node (*i*), a penalised logistic regression was fitted with *i* as the outcome and the remaining items (*j*) as predictors. A one-standard-error penalty was selected from 10-fold cross-validation. An undirected edge (i.e. adjusted association between two nodes) between items *i* and *j* was retained only if both directional models selected the other item (AND-rule). Model performance was evaluated using cross-validated area under the curve (AUC), binomial deviance, and the number of predictors selected at the one-standard-error penalty. Logistic regression models were then conducted for each retained edge, adjusted for all other items. For interpretation, symmetrised adjusted ORs were calculated by averaging the two directional conditional ORs. Analyses were conducted using IBM SPSS version 29 (IBM Corp., 2022) and R version 4.4.3 (R Core Team, 2025) using “survey” (Lumley, 2024), “psych” (Revelle, 2025), “qgraph” (Epskamp et al., 2012), “glmnet” (Tay et al., 2023), and “ggplot2” packages (Wickham, 2016).

## Results

Among the pooled sample of 4,918 men, 8.0% (99% CI [6.9%, 9.2%]) were sexually attracted to children, 7.4% ([6.4%, 8.6%]) would very likely or definitely have sexual contact with a child aged 14 or under if it went undetected, 5.7% ([4.8%, 6.8%]) were concerned about their sexual feelings towards children, 5.7% ([4.7%, 6.8%]) would watch a webcam sex show of a child if offered, and 5.5% ([4.6%, 6.6%]) would watch CSAM online if anonymity were guaranteed. By comparison, the prevalence of sexual behaviours towards children by adult men were around half as common: 4.7% ([3.8%, 5.6%]) flirted with children online, 4.6% ([3.8%, 5.6%]) had sexual contact with a child, 3.5% ([2.8%, 4.3%]) intentionally viewed CSAM, 2.7% ([2.1%, 3.5%]) purchased sexual interactions with children or CSAM online, and 2.4% ([1.9%, 3.1%]) engaged in a sexual webcam with a child. The unweighted and weighted frequencies are shown in Supplemental Table 1.

### Country-Specific Prevalence Estimates

Table 1 shows clear between-country differences in the prevalence of 9 of 10 measures of men’s sexual behaviours, feelings, and interests towards children (adjusted Rao–Scott F tests). Across all items, U.S. men (range 4.2%–9.9%) reported higher prevalence than men in Australia (range 1.7%–7.4%) or the United Kingdom (range 1.4%–7.0%), who were generally similar. Illustratively, United States estimates exceeded Australia and the United Kingdom for intentionally viewing CSAM (5.2% vs. 2.5%–3.0%), sexual contact with a child (6.1% vs. 3.2%–4.9%), willingness to view CSAM (7.4% vs. 4.1%–5.2%), sexual feelings towards children (9.7% vs. 7.0%–7.4%), and likely sexual contact with a child under 14 (9.9% vs. 5.9%–6.7%). The largest relative gaps were for concern about sexual feelings (United States = 8.8% vs. 4.3%–4.5%; *F* = 13.76, *p* < .001), purchasing CSAM online (4.8% vs. 1.7%–2.0%; *F* = 13.04, *p* < .001), and sexually webcammed a child (4.2% vs. 1.4%–1.8%; *F* = 12.01, *p* < .001), each roughly two to three times higher in the United States. The only exception was willingness to watch a webcam sex show, which did not significantly differ by country (5.3%–6.0%; *F* *=* 1.98, *p* = .726).

**Table 1.** Weighted Prevalence (99% CI) of Sexual Behaviours, Feelings, and Interests by Country.

	Australia (*n* = 1,939)	United Kingdom (*n* = 1,506)	United States (*n* = 1,473)
Measures	% (99% CI)	% (99% CI)	% (99% CI)
Intentionally viewed CSAM	2.5% (1.7%, 3.8%)	3.0% (1.9%, 4.7%)	5.2% (3.7%, 7.2%)
	*F*(2,9813.71) = 7.01, *p* < .001		
Sexual contact with child	3.2% (2.2%, 4.7%)	4.9% (3.4%, 6.9%)	6.1% (4.5%, 8.1%)
	*F*(2,9844.86) = 6.10, *p* = .002		
Flirted with children online	4.3% (3.1%, 6.0%)	3.8% (2.5%, 5.6%)	6.0% (4.4%, 8.0%)
	*F*(2,9841.79) = 3.30, *p* = .037		
Webcammed child in sexual way	1.8% (1.1%, 2.9%)	1.4% (0.8%, 2.6%)	4.2% (2.9%, 6.1%)
	*F*(2,9846.78) = 12.01, *p* < .001		
Purchased CSAM online	1.7% (1.0%, 2.8%)	2.0% (1.2%, 3.5%)	4.8% (3.4%, 6.8%)
	*F*(2,9843.84) = 13.04, *p* < .001		
Would view CSAM	4.1% (3.0%, 5.6%)	5.2% (3.7%, 7.2%)	7.4% (5.6%, 9.8%)
	*F*(2,9831.78) = 6.78, *p* < .001		
Would watch webcam sex show	5.3% (3.8%, 7.3%)	5.9% (4.2%, 8.2%)	6.0% (4.4%, 8.0%)
	*F*(2,9772.60) = 1.98, *p* = .726		
Sexual feelings towards children	7.4% (5.7%, 9.5%)	7.0% (5.3%, 9.2%)	9.7% (7.7%, 12.1%)
	*F*(2,9821.89) = 3.34, *p* = .036		
Concerned about sexual feelings	4.5% (3.3%, 6.2%)	4.3% (3.0%, 6.1%)	8.8% (6.9%, 11.2%)
	*F*(2,9847.45) = 13.76, *p* < .001		
Likely sexual contact under 14	6.7% (5.2%, 8.6%)	5.9% (4.3%, 8.0%)	9.9% (7.9%, 12.4%)
	*F*(2,9849.51) = 7.77, *p* < .001		

*Note.* Tests of independence based on adjusted *F*, a variant of the second-order Rao–Scott adjusted chi-squared statistic. CSAM = child sexual abuse material.

### Age Distribution

**Figure 1.**
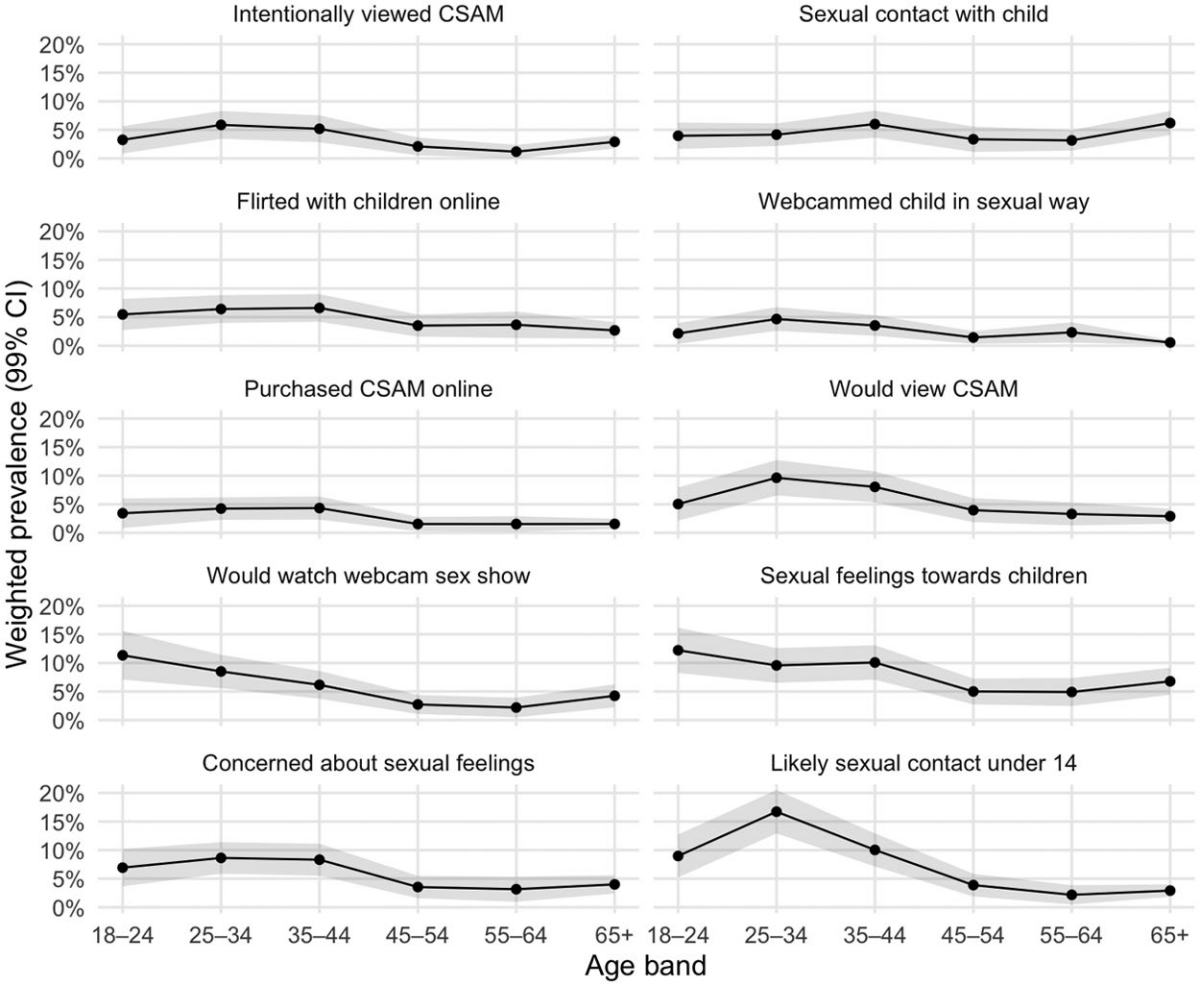
Weighted prevalence (99% CI) of sexual behaviours, feelings, and interests by age bands (*N* = 4,918).

Figure 1 displays the proportion of men within each age-band reporting sexual behaviours, feelings, and interests towards children; exact percentages are provided in Supplemental Table 2. Across most items, prevalence was highest in early adulthood and declined with age. Peaking at age 25 to 34 years were intentional CSAM viewing (5.9%), willingness to view CSAM (9.6%), webcamming child in a sexual way (4.7%), likely sexual contact with child 14 and under (16.7%), and concern about sexual feelings towards children (8.6%). Sexual feelings towards children and willingness to watch a webcam sex show were highest at 18 to 24 years (12.2% and 11.3%) and reduced thereafter (2.2% and 4.9% at 55–64 years), followed by an uptick at age 65 years and older (6.8% and 4.2%, respectively). Flirting with children online and purchasing CSAM online were concentrated at ages 25 to 44 years (6.4%–6.6% and 4.2%–4.3%, respectively), with prevalence halved at age 45 and older (2.7%–3.5% and 1.5%–1.6%, respectively). By comparison, sexual contact with children spiked at ages 35 to 44 (6.0%) and 65 years and older (6.2%).

### Demographic Correlates

Table 2 presents the odds of endorsing the sexual behaviour, feelings, and interest measures across six demographic indicators of socio-economic status and trust. Notably, all indicators were associated with significantly greater odds of intentionally viewing CSAM (range OR = 1.99–4.49), webcamming a child in a sexual way (range OR = 2.09–6.35), concern about sexual feelings towards children (range OR = 1.67–3.23), and likely sexual contact with child under 14 (range OR = 1.46–3.55). The largest significant effect sizes were for men who work with children, followed by those who lived with one or more child. Specifically, these men were 6.35 and 4.70 times as likely to have webcammed a child in a sexual way, 5.64 and 4.14 times as likely to purchase CSAM online, 4.49 and 2.88 times as likely to intentionally watch CSAM, 3.55 and 3.73 times as likely to have sexual contact with a child under 14, and 3.23 and 2.44 times as likely to be concerned about their sexual feelings towards children, respectively.

**Table 2.** Odds (99% CI) of Sexual Behaviours, Feelings, and Interests by Demographic Indicators of Socio-economic Status and Trust (*N* = 4,918).

	High household income	Married or de facto	Employed	Bachelor’s degree or higher	Child in household	Works with children
Measures	OR (99% CI)	OR (99% CI)	OR (99% CI)	OR (99% CI)	OR (99% CI)	OR (99% CI)
Intentionally view CSAM	2.48 (1.55, 3.95)	2.12 (1.19, 3.76)	2.84 (1.46, 5.50)	1.99 (1.24, 3.18)	2.88 (1.80, 4.61)	4.49 (2.81, 7.20)
Sexual contact with child	1.60 (1.03, 2.48)	1.37 (0.89, 2.10)	1.04 (0.68, 1.59)	1.32 (0.88, 1.99)	1.79 (1.19, 2.68)	2.36 (1.51, 3.69)
Flirted with children online	1.58 (1.04, 2.41)	1.13 (0.73, 1.73)	1.89 (1.13, 3.18)	1.67 (1.11, 2.50)	2.31 (1.54, 3.47)	2.80 (1.84, 4.26)
Webcammed child in sexual way	2.50 (1.44, 4.31)	2.09 (1.06, 4.14)	4.29 (1.67, 11.02)	2.64 (1.50, 4.65)	4.70 (2.63, 8.41)	6.35 (3.66, 11.00)
Purchased CSAM online	2.07 (1.24, 3.45)	1.58 (0.87, 2.87)	2.16 (1.05, 4.44)	1.91 (1.13, 3.22)	4.14 (2.34, 7.30)	5.64 (3.34, 9.52)
Would view CSAM	1.39 (0.93, 2.08)	1.45 (0.95, 2.21)	1.52 (0.96, 2.41)	1.60 (1.10, 2.34)	2.03 (1.39, 2.96)	3.35 (2.27, 4.95)
Would watch webcam sex show	0.98 (0.64, 1.51)	0.98 (0.65, 1.47)	1.63 (1.00, 2.66)	1.37 (0.93, 2.02)	1.90 (1.29, 2.80)	2.63 (1.76, 3.93)
Sexual feelings towards children	1.37 (0.99, 1.92)	0.81 (0.59, 1.12)	1.19 (0.84, 1.69)	1.47 (1.07, 2.02)	1.35 (0.98, 1.86)	2.08 (1.47, 2.94)
Concerned about sexual feelings	1.97 (1.35, 2.86)	1.67 (1.09, 2.57)	1.72 (1.08, 2.72)	1.74 (1.21, 2.52)	2.44 (1.68, 3.54)	3.23 (2.22, 4.69)
Likely sexual contact under 14	2.33 (1.68, 3.25)	1.73 (1.20, 2.49)	3.27 (1.95, 5.49)	1.46 (1.06, 2.02)	3.73 (2.67, 5.22)	3.55 (2.53, 4.96)

*Note.* CSAM = child sexual abuse material.

### Overlap Across Sexual Behaviours, Feelings, and Interests

Table 3 displays the row proportion of overlap across the sexual behaviours, feelings, and interest measures (percentages based on column numerator and row denominator) with the odds of item co-endorsement presented underneath. Effect sizes were substantial, ranging from 4.80 to 88.37, and all statistically significant at *p* < .01. Row-wise overlaps show strong co-occurrence of online behaviours. Among those who had webcammed a child (*n* = 117), most had also flirted with children online (73.0%; OR = 88.37), purchased CSAM online (55.9%; OR = 87.47), were willing to view CSAM (59.1%; OR = 33.30), had sexual feelings towards children (58.7%; OR = 19.51), and would likely have sexual contact with a child under 14 (59.9%; OR = 22.93). Similarly, among those who had purchased CSAM online (*n* = 134), two-thirds indicated likely sexual contact under 14 (66.2%; OR = 32.15), and about half reported sexual feelings (54.4%; OR = 16.55), concern about feelings (50.4%; OR = 21.41), and online flirting (50.4%; OR = 29.20). Men who intentionally viewed CSAM (*n* = 169) also often endorsed likely sexual contact under 14 (58.1%; OR = 23.44), concern about sexual feelings (44.0%; OR = 17.02), willingness to watch a webcam show (43.2%; OR = 16.80), and had sexual contact with a child (39.7%; OR = 19.13).

**Table 3.** Overlap (Row Proportions) and Co-Endorsement Odds of Sexual Behaviours, Feelings, and Interests (*N* = 4,918).

Measures	*n*	1	2	3	4	5	6	7	8	9	10
1. Intentionally view CSAM	169	–	39.7% [19.13]	27.0% [9.24]	23.6% [18.40]	25.4% [17.24]	38.9% [14.25]	43.2% [16.80]	35.3% [7.20]	44.0% [17.02]	58.1% [23.44]
2. Sexual contact with child	223	30.0% [19.13]	–	20.1% [6.20]	15.0% [9.56]	14.0% [7.23]	19.5% [4.81]	26.5% [7.35]	26.9% [4.80]	27.8% [7.74]	40.4% [11.00]
3. Flirted with children online	226	20.2% [9.24]	19.9% [6.20]	–	37.8% [88.37]	29.7% [29.20]	34.8% [12.64]	29.2% [8.70]	40.5% [9.85]	31.5% [9.71]	36.0% [8.81]
4. Webcammed child in sexual way	117	34.0% [18.40]	28.5% [9.56]	73.0% [88.37]	–	55.9% [87.47]	59.1% [33.30]	48.4% [19.28]	58.7% [19.51]	50.1% [20.37]	59.9% [22.93]
5. Purchased CSAM online	134	32.1% [17.24]	23.4% [7.23]	50.4% [29.20]	49.2% [87.47]	–	50.0% [22.65]	44.5% [16.66]	54.4% [16.55]	50.4% [21.41]	66.2% [32.15]
6. Would view CSAM	267	24.6% [14.25]	16.4% [4.81]	29.5% [12.64]	26.0% [33.30]	25.0% [22.65]	–	34.2% [12.39]	36.0% [8.22]	28.7% [12.39]	36.3% [9.37]
7. Would watch webcam	277	26.4% [16.80]	21.4% [7.35]	23.9% [8.70]	20.5% [19.28]	21.5% [16.66]	33.0% [12.39]	–	38.7% [9.58]	33.6% [11.79]	37.9% [10.38]
8. Sexual feelings towards children	391	15.3% [7.20]	15.4% [4.80]	23.4% [9.85]	17.6% [19.51]	18.6% [16.55]	24.6% [8.22]	27.4% [9.58]	–	21.4% [5.96]	25.5% [5.54]
9. Concerned about sexual feelings	281	26.4% [17.02]	22.0% [7.74]	25.4% [9.71]	20.9% [20.37]	23.9% [21.41]	27.2% [12.39]	33.0% [11.79]	29.7% [5.96]	–	44.3% [14.72]
10. Likely sexual contact under 14	360	27.2% [23.44]	25.1% [11.00]	22.6% [8.81]	19.5% [22.93]	24.6% [32.15]	26.9% [9.37]	29.1% [10.38]	27.7% [5.54]	34.7% [14.72]	–

*Note.* ORs based on total sample and presented in square brackets. All ORs significant at *p* < .01. CSAM = child sexual abuse material.

In contrast, items regarding sexual feelings and interests towards children showed more moderate overlap. Men willing to watch a webcam show most often also reported sexual feelings towards children (38.7%; OR = 9.58), likely sexual contact of child under 14 (37.9%; OR = 10.38), concern about feelings (33.6%; OR = 11.79), and willingness to watch CSAM (33.0%; OR = 12.39). Those willing to view CSAM if anonymous most commonly co-endorsed likely sexual contact with a child under 14 (36.3%; OR = 9.37), sexual feelings towards children (36.0%; OR = 8.22), and willingness to watch webcam sex show (34.2%; OR = 12.39). Sexual contact with a child showed lower overlaps overall, with its highest co-endorsement being likely sexual contact with a child under 14 (40.4%; OR = 11.00) and intentionally viewing CSAM (30.0%; OR = 19.13).

### Conditional Dependence Network

Nodewise LASSO with 10-fold cross-validation produced a conditional dependence network with 24 Ising edges (i.e. direct adjusted relationship) out of a possible 45. Variable selection was based on the AND-rule, meaning an undirected link between two items is retained only when each item is independently associated with the other after accounting for all remaining items; one-sided links are discarded, and the retained link’s strength is taken as the average of the two directional effects. After applying the AND-rule, each item’s LASSO model at the one-standard-error penalty retained 5.2 of the 9 possible associations on average. Predictive performance was good (mean AUC = 0.81, interquartile range = 0.76–0.86) but varied across items.

The highest discrimination was observed for webcammed a child in a sexual way (AUC = 0.95; deviance = 0.11; 4 predictors selected) and bought CSAM online (AUC = 0.90, deviance = 0.15; 6 predictors). Intermediate performance was seen for intentionally viewed CSAM (AUC = 0.88; deviance = 0.21; 5 predictors), willing to watch webcam sex show (AUC = 0.80; deviance = 0.36; 7 predictors), and flirted with children online (AUC = 0.80; deviance = 0.29; 4 predictors), concern about sexual feelings towards children (AUC = 0.79; deviance = 0.37; 5 predictors), and likely sexual contact with child under 14 (AUC = 0.77; deviance = 0.41; 7 predictors). The items with the worst performance were sexual feelings towards children (AUC = 0.70; deviance = 0.49; 5 predictors) and sexual contact with a child (AUC = 0.73; deviance = 0.33; 2 predictors).

**Figure 2.**
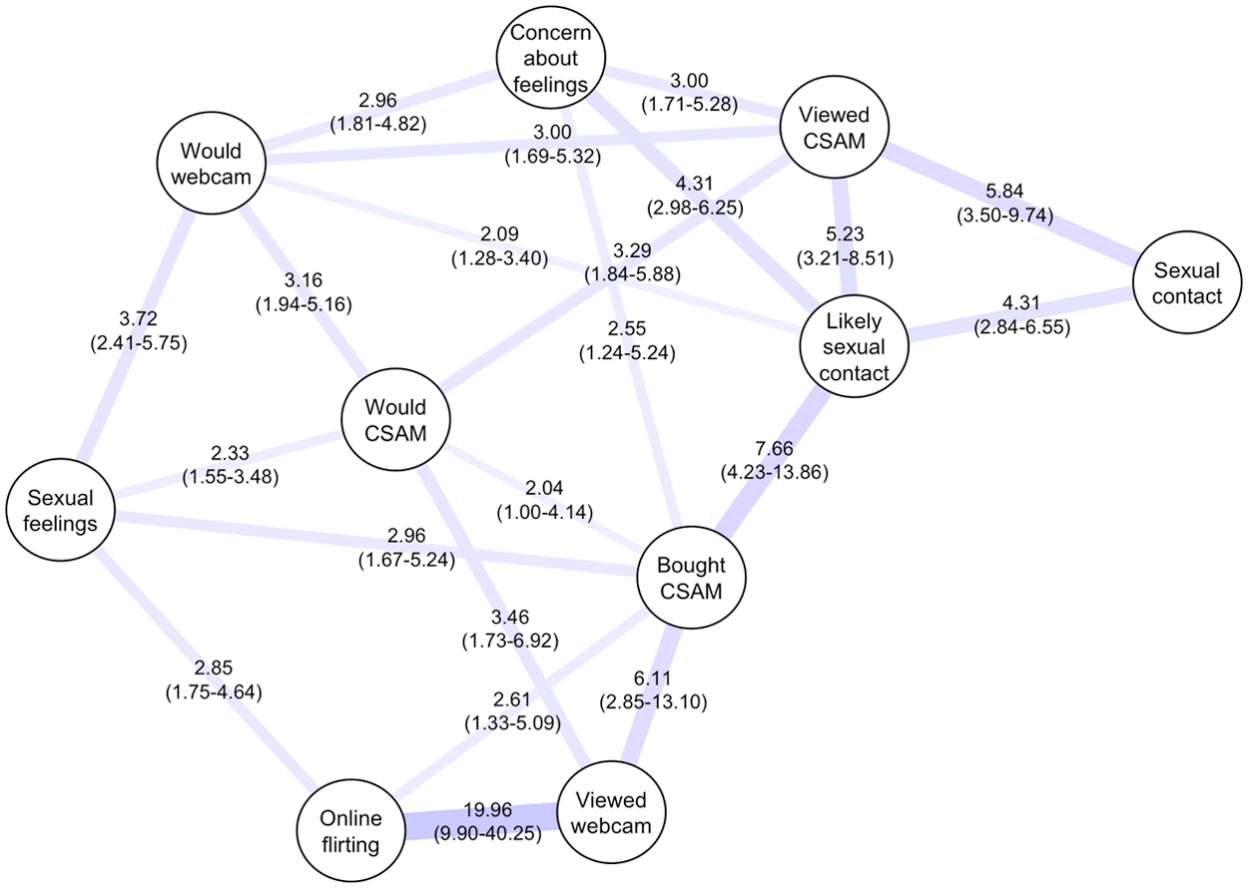
Conditional dependence network of sexual behaviours, feelings, and interest items (symmetrised mean conditional OR [99% CI]).

Figure 2 presents the conditional dependence network with symmetrised mean conditional ORs (99% CI). This diagram shows the associations between items after accounting for all other relevant items. Each node (circle) represents an item. Lines (Ising edges) linking each node indicate a direct adjusted association, with line thickness corresponding to the strength of this relationship. Each edge is overlaid with the adjusted symmetrised conditional OR (99% CI) to reflect a single undirected association.

The strongest adjusted associations appeared within a coherent cluster of online behaviours: flirting with children online and viewing sexual webcams (OR = 19.96), bought CSAM online and likely sexual contact with a child under 14 (OR = 7.66), viewed webcam and bought CSAM online (OR = 6.1), intentionally viewed CSAM and sexual contact with a child (OR = 5.84), and viewed CSAM and likely sexual contact (OR = 5.23). A cluster on sexual feelings and interests was also evident: willing to watch CSAM (CSAM interest) and willing to watch sexual webcam (webcam interest; OR = 3.16), webcam interest and sexual feelings towards children (OR = 3.72), and concern about feelings and sexual contact interest (OR = 4.31). Overall, the pattern suggests two interlinked trends of co-endorsement: one running from online grooming through webcam viewing towards purchasing CSAM and sexual contact interest, and another from webcam and CSAM interest to viewing CSAM and sexual contact.

## Discussion

This study provides cross-country estimates of men’s self-reported sexual behaviours, feelings, and interests towards children, together with age distributions, demographic correlates, co-endorsement, and conditional dependence among 10 indicators. Using large, weighted quota-based samples of men from Australia, the United Kingdom, and the United States, we show that markers of sexual interest are more prevalent than enacted behaviours, that the United States exhibits consistently higher levels than Australia and the United Kingdom across most items, and that co-endorsement patterns are structured and interpretable. The study’s novelty lies in its multi-country design; its simultaneous treatment of prevalence, age gradients, and sociodemographic variation; and its use of a conditional dependence network to map adjusted co-endorsement across multiple indicators. Collectively, the findings provide comparable baseline estimates and an empirical account of how interests and behaviours cluster in the community, informing prevention and early-identification strategies.

Consistent with the extant literature, prevalence estimates followed a clear gradient, where feelings and interest were more common than self-reported behaviours (Bártová et al., 2021; Dombert et al., 2016; Joyal & Carpentier, 2017; Seto et al., 2015). Across all participants, indicators of sexual feelings and likely contact if undetected were reported by a small but notable minority, whereas deliberate CSAM viewing, online sexualised interactions, purchasing CSAM, and sexual contact were rarer. The same pattern was observed separately for each country. Importantly, the proportions endorsing these items were consistently higher in the United States than in the United Kingdom or Australia, a finding consistent with prior cross-country research (Ó Ciardha et al., 2022). A potential explanation may be cross‑national differences in opportunity structures and online ecosystems, such as greater exposure to high‑risk platforms, lower perceived detection risk, or higher access to children through work or household roles. Practically, these estimates underscore the need for tiered prevention that distinguishes between interest and behaviour, expanding low-barrier pathways to confidential support for men who report sexual feelings or concern while maintaining robust disruption and enforcement capacity for offending.

Age patterns were consistent and interpretable. Most indicators peaked in early adulthood and declined thereafter, with some late-life upticks for sexual feelings and contact. Interest measures were highest among young men, with online behaviours clustered in early to mid-adulthood. Sexual contact with children showed a later peak with a possible second rise at older ages. This non-linear trend has been observed in other studies (Napier et al., 2024; Seigfried-Spellar, 2014). These gradients suggest age-specific opportunity structures and self-regulation dynamics. For example, early adulthood may be a critical window for primary prevention and demand-reduction messaging, whereas older-age peaks in selected behaviours caution against exclusively youth-focused strategies. Methodologically, modelling age non-linearly and presenting age-banded estimates provided nuanced insight. Future longitudinal or cohort-sequential designs could clarify whether these patterns reflect ageing, period effects, or cohort differences.

Associations with sociodemographic indicators of social trust and access were robust and directionally consistent. Higher household income, being partnered, having children in the household, employment, university education, and working with children were each associated with greater odds of endorsing several items, with the largest effects typically observed for household and work-related access to children. These patterns are compatible with opportunity-based explanations: roles connoting trust or providing contact may both facilitate offending and reduce detection risk (Seto, 2019; Wortley & Smallbone, 2014). Policy implications include targeted safeguarding in occupational and volunteer settings, strengthened screening and supervision for child-contact roles, and routine reinforcement of professional boundaries. Research should probe mechanisms (e.g. opportunity structures, guardianship, and self-selection into child-contact roles) and test whether the observed associations persist when adjusting for psychosocial covariates such as pornography use, mental health, and adverse childhood experiences.

Co-endorsement analyses revealed non-random clustering. Online behaviours co-occurred at high rates and were frequently paired with interest measures and with likely sexual contact if undetected, whereas sexual feelings and concerns showed broader but more moderate overlaps. Sexual contact with children exhibited lower overall overlap but was strongly associated with viewing CSAM and with likely contact. Practically, these patterns support risk screening that integrates multiple indicators rather than treating each in isolation. They also point to differentiated intervention pathways. For example, men endorsing interest-only profiles may benefit most from confidential therapeutic supports and self-management tools, while those reporting online behaviours warrant both clinical support and situational controls to reduce access and opportunity.

The conditional dependence network distilled these bivariate patterns into adjusted, direct associations and identified three interpretable clusters: (a) online offending (i.e. online flirting, sexual webcamming, deliberate CSAM viewing, purchasing CSAM); (b) contact offending; and (c) interest in offending (i.e. sexual feelings, willingness to view CSAM or watch a sexual webcam show, concern about feelings, and likely contact if undetected). The network suggests partially distinct yet connected pathways, with strong within-cluster ties and bridging links between interest and online behaviours and between online behaviours and contact outcomes. This structure has practical value. For example, prevention and treatment may be optimised by segmenting men into cluster-aligned profiles with tailored supports, such as anonymous help-seeking channels for interest-dominant profiles, access-control and offence-pathway interruption for online-dominant profiles, and intensive multi-modal responses for contact-linked profiles.

The present findings motivate several next steps using these data. First, we will examine demographic and internet-use characteristics associated with online offending to differentiate profiles within the online cluster. Second, we will assess cross-country differences in adverse childhood experiences among men endorsing online offending and interest, testing whether patterns of adverse childhood experiences help explain between-country prevalence gaps. Third, we will analyse attitudes towards CSEA to determine how these align with interest, online, and contact offending. Fourth, we will identify factors that differentiate men who report sexual interests only, sexual contact only, and both interest and contact, clarifying potential interest and offender profiles. Fifth, we will compare men who do and do not express a desire for help regarding their sexual feelings towards children, to guide design of low-friction support pathways. Each thread responds directly to the current study’s evidence of cluster-specific patterns and the policy need to distinguish interest-dominant from behaviour-dominant profiles.

This study has several notable strengths, including its large quota-matched samples across three countries, post-stratification weights, and design-appropriate inferences. However, the findings of this study must be interpreted within the context of its limitations. Foremost, self-reports of highly stigmatised behaviours may be biased despite anonymity; while our quality controls and dishonesty screens mitigate this risk, residual misreporting is possible. Second, cross-sectional data preclude causal inference and cannot isolate age, period, and cohort effects. Third, while quota sampling with post-stratification improves representativeness, unmeasured selection cannot be ruled out. Finally, the sample included only males, limiting insights into female sexual interests and behaviours towards children, an area that remains underexplored in the literature. Future work should triangulate with longitudinal designs, administrative linkages (where ethical and lawful), and targeted qualitative inquiry to deepen understanding.

## Conclusion

Using large, weighted, quota-based samples of adult men from Australia, the United Kingdom, and the United States, this study provides comparable, population-based estimates of men’s sexual feelings, interests, and behaviours towards children; maps age gradients and sociodemographic correlates linked to trust and access; and identifies coherent clusters of co-endorsement separating interest, online offending, and contact offending. The results provide some support for tiered prevention that distinguishes interest from behaviour, age-responsive strategies, and targeted safeguards in child-contact contexts, while highlighting the value of anonymous, community-based surveys to reveal undetected interests and behaviours. Although the self-report and cross-sectional design limit causal inference and may leave residual misreporting, the integrated prevalence, correlational, and network evidence offers a pragmatic foundation for policy and practice and motivates the planned programme of analyses on online-offending profiles, adversity, attitudes, pathways, and help-seeking to refine prevention and intervention.

## Supplemental Material

sj-docx-1-jiv-10.1177_08862605251403602 – Supplemental material for Exploring Patterns of Men’s Self-Reported Sexual Behaviours, Feelings, and Interests Towards Children Supplemental material, sj-docx-1-jiv-10.1177_08862605251403602 for Exploring Patterns of Men’s Self-Reported Sexual Behaviours, Feelings, and Interests Towards Children by Tyson Whitten, Michael Salter and Delanie Woodlock in Journal of Interpersonal Violence
