# Supplementary material for: Exploring Patterns of Men’s Self-Reported Sexual Behaviours, Feelings, and Interests Towards Children
Source: J Interpers Violence. 2026 Mar 3;41(7-8):1491–513. doi: 10.1177/08862605251403602 (PMC12960736; doi:10.1177/08862605251403602)
Supplement: sj-docx-1-jiv-10.1177_08862605251403602 – Supplemental material for Exploring Patterns of Men’s Self-Reported Sexual Behaviours, Feelings, and Interests Towards Children [file sj-docx-1-jiv-10.1177_08862605251403602.docx]

| Supplementary table 1. Descriptive statistics pooled sample (N = 4,918) | | | |
| --- | --- | --- | --- |
|  | Unweighted  n | Weighted | |
|  |  | n | % (99% CI) |
| Age |  |  |  |
| 18 – 24 years | 555 | 638 | 13.0% (11.5% - 14.6%) |
| 25 – 34 years | 937 | 866 | 17.6% (16.1% - 19.3%) |
| 35 – 44 years | 858 | 834 | 17.0% (15.4% - 18.6%) |
| 44 – 54 years | 753 | 796 | 16.2% (14.6% - 17.9%) |
| 55 – 64 years | 730 | 766 | 15.6% (14.1% - 17.2%) |
| 65 years and older | 1,094 | 1,018 | 20.7% (19.0% - 22.5%) |
| High annual household income | 1,363 | 1,146 | 23.3% (21.6% - 25.2%) |
| Married or de facto relationship | 3,206 | 2,951 | 60.0% (57.8% - 61.2%) |
| Full/part time or casually employed | 3,430 | 3,229 | 65.6% (63.5% - 67.7%) |
| Bachelor’s degree or higher | 1,923 | 1,838 | 37.4% (35.3% - 39.5%) |
| One or more child lives in household | 1,735 | 1,603 | 32.6% (30.6% - 34.6%) |
| Work involves contact with children | 899 | 792 | 16.1% (14.6% - 17.7%) |
| Intentionally view CSAM | 199 | 169 | 3.5% (2.8% - 4.3%) |
| Sexual contact with child | 245 | 223 | 4.6% (3.8% - 5.6%) |
| Flirted with children online | 247 | 226 | 4.7% (3.8% - 5.6%) |
| Webcammed child in sexual way | 135 | 117 | 2.4% (1.9% - 3.1%) |
| Purchased CSAM online | 149 | 134 | 2.7% (2.1% - 3.5%) |
| Would view CSAM | 277 | 267 | 5.5% (4.6% - 6.6%) |
| Would watch webcam sex show | 279 | 277 | 5.7% (4.7% - 6.8%) |
| Sexual feelings towards children | 407 | 391 | 8.0% (6.9% - 9.2%) |
| Concerned about sexual feelings | 310 | 281 | 5.7% (4.8% - 6.8%) |
| Likely sexual contact under 14 | 413 | 360 | 7.4% (6.4% - 8.6%) |

| Supplementary table 2. Weighted prevalence (99% CI) of sexual behaviours, feelings, and interests by age bands (N = 4,918). | | | | | | |
| --- | --- | --- | --- | --- | --- | --- |
|  | 18 – 24 years | 25 – 34 years | 35 – 44 years | 45 – 54 years | 55 – 64 years | 65 years and older |
|  | n = 638 | n = 866 | n = 834 | n = 796 | n = 766 | n = 1,018 |
| Intentionally view CSAM | 3.3%  (1.6% - 6.7%) | 5.9%  (3.9% - 8.8%) | 5.2%  (3.3% - 8.1%) | 2.1%  (1.0% - 4.4%) | 1.2%  (0.4% - 3.2%) | 2.9%  (1.9% - 4.3%) |
| Sexual contact with child | 4.0%  (2.2% - 7.1%) | 4.2%  (2.6% - 6.7%) | 6.0%  (4.0% - 8.9%) | 3.4%  (1.7% - 6.4%) | 3.2%  (1.8% - 5.5%) | 6.2%  (4.4% - 8.7%) |
| Flirted with children online | 5.5%  (3.3% - 8.9%) | 6.4%  (4.4% - 9.3%) | 6.6%  (4.6% - 9.5%) | 3.5%  (2.0% - 6.0%) | 3.7%  (1.9% - 6.8%) | 2.7%  (1.6% - 4.6%) |
| Webcammed child in sexual way | 2.1%  (0.9% - 5.0%) | 4.7%  (3.0% - 7.2%) | 3.5%  (2.1% - 5.9%) | 1.4%  (0.7% - 3.1%) | 2.3%  (1.1% - 5.0%) | 0.6%  (0.3% - 1.2%) |
| Purchased CSAM online | 3.4%  (1.6% - 7.2%) | 4.2%  (2.7% - 6.7%) | 4.3%  (2.7% - 6.9%) | 1.5%  (0.7% - 3.4%) | 1.5%  (0.6% - 3.7%) | 1.6%  (0.9% - 2.8%) |
| Would view CSAM | 5.0%  (2.8% - 8.7%) | 9.6%  (6.9% - 13.2%) | 8.0%  (5.7% - 11.1%) | 3.9%  (2.3% - 6.6%) | 3.3%  (1.8% - 5.9%) | 2.9%  (1.8% - 4.5%) |
| Would watch webcam sex show | 11.3%  (7.7% - 16.3%) | 8.5%  (6.0% - 11.9%) | 6.2%  (4.1% - 9.1%) | 2.7%  (1.5% - 5.0%) | 2.2%  (1.0% - 4.8%) | 4.2%  (2.6% - 6.8%) |
| Sexual feelings towards children | 12.2%  (8.8% - 16.8%) | 9.6%  (6.9% - 13.1%) | 10.1%  (7.4% - 13.5%) | 5.0%  (3.2% - 7.8%) | 4.9%  (3.0% - 8.0%) | 6.8%  (4.8% - 9.6%) |
| Concerned about sexual feelings | 6.9%  (4.3% - 11.0%) | 8.6%  (6.2% - 11.8%) | 8.3%  (5.9% - 11.6%) | 3.5%  (2.0% - 6.1%) | 3.2%  (1.6% - 6.3%) | 4.0%  (2.7% - 5.9%) |
| Likely sexual contact under 14 | 9.0%  (5.9% - 13.5%) | 16.7%  (13.3% - 20.9%) | 10.0%  (7.5% - 13.3%) | 3.9%  (2.3% - 6.4%) | 2.2%  (1.0% - 4.7%) | 2.9%  (2.0% - 4.2%) |
